# Emerging Biomarkers of Oxidative Stress in Acute and Stable Coronary Artery Disease: Levels and Determinants

**DOI:** 10.3390/antiox8050115

**Published:** 2019-05-01

**Authors:** Valter Lubrano, Alessandro Pingitore, Irene Traghella, Simona Storti, Serena Parri, Sergio Berti, Rudina Ndreu, Andrea Andrenelli, Cataldo Palmieri, Giorgio Iervasi, Francesca Mastorci, Cristina Vassalle

**Affiliations:** 1 Fondazione CNR-Regione Toscana G Monasterio, 56100 Pisa, Italy; walterl@ftgm.it (V.L.); itraghella@ftgm.it (I.T.); 2 Istituto di Fisiologia Clinica, CNR, 56100 Pisa, Italy; pingi@ifc.cnr.it (A.P.); rudina.ndreu@ifc.cnr.it (R.N.); iervasi@ifc.cnr.it (G.I.); mastorcif@ifc.cnr.it (F.M.); 3 Ospedale del Cuore G Pasquinucci, Fondazione CNR-Regione Toscana G Monasterio, 54100 Massa, Italy; storti@ftgm.it (S.S.); parri@ftgm.it (S.P.); berti@ftgm.it (S.B.); andrenelli@ftgm.it (A.A.); palmieri@ftgm.it (C.P.)

**Keywords:** oxidative stress, acute coronary syndrome, stable coronary artery disease, biomarkers

## Abstract

Background: Oxidative stress is crucial in the pathogenesis of atherosclerosis and acute myocardial infarction (AMI). Under the generic terms “oxidative stress” (OS), many biomarkers belonging to different pathways have been proposed. Aim: To compare the levels of recently proposed OS-related parameters in acute coronary syndromes (ACS) and stable coronary artery disease (CAD), to evaluate their effectiveness as additive risk or illness indicators of stable and acute ischemic events, and their response over time during the course of AMI. Methods: 76 ACS, 77 CAD patients, and 63 controls were enrolled in the study. Different OS-related biomarkers, including reactive oxygen metabolites (ROM), the total antioxidant capacity (OXY), nitrite/nitrate (final nitric oxide products, NO_x_), and Lectin-like oxidized low-density lipoprotein receptor-1 (LOX-1), were evaluated. Moreover, time response during AMI course (admission, and 6, 12, 18, 24, 36, and 48 hours after, T0-T6, respectively) and correlation with traditional cardiovascular (CV) risk factors (age, gender, hypertension, diabetes mellitus, dyslipidemia, smoking habit) were also assessed. Results: Over time, ROM progressively increased while OXY and NO_x_ decreased. Kinetics of LOX-1 during AMI shows that this biomarker boosts early during the acute event (T1 and T2) and then progressively decreases, being significantly lower from T0 to T6. Different OS-related biomarkers were differentially associated with CV risk factors and CAD or ACS presence. Conclusion: Differences in OS-related biomarkers (between groups, according to the response over time during AMI, and to the presence of CV risk factors) confirmed OS involvement in the transition from healthy status to stable CAD and ACS, although evidencing the heterogeneous nature of redox processes. In future, a multi-marker panel including different biomarkers and pathways of oxidative stress could be evaluated as an additive tool to be used in the CV prevention, diagnosis, patient stratification, and treatment.

## 1. Introduction

Oxidative stress (increased oxidant generation with impairment of endogenous antioxidant mechanisms) plays an important role in the pathogenesis of atherosclerosis, and it is increased by traditional cardiovascular (CV) risk factors (e.g., diabetes mellitus, dyslipidemia, smoking, gender, and age) [1,2,3]. 

Assuming that traditional risk factors explain a large proportion of CV risk, they can be ineffective in some cases, because they are unable to explain why some high-risk patients did not experience a CV event, even in the long term, while a percentage of patients classified as low risk did (e.g., with none or only one of the traditional CV risk factor) [4]. These patients may be better classified by using alternative/additional biomarkers, giving targeted and appropriate management to those who would need more [5]. Nonetheless, at the moment, the gain of a single biomarker over the predictive power estimated with traditional risk factors is generally found to be limited [5]. Conversely, it is important to mention that the discrimination power can be further improved when biomarkers with a low degree of correlation, and as such belonging to independent pathways, thus reflecting different pathophysiological events, are considered [5]. This concept may be true also for the oxidative stress, where many oxidative stress-related biomarkers have been proposed, involving both the oxidative and antioxidant counterparts, which reflect the many different pathways that the generic term “oxidative stress” included [4]. Moreover, whether there are a number of common biological aspects between stable coronary artery disease (CAD) and acute coronary syndromes (ACS), there are also differences between these two clinical situations. Therefore, biomarkers reflecting various oxidative-related pathways may vary in chronic CAD and ACS patients. Among the many biomarkers of oxidative stress recently proposed, we considered some oxidative stress biomarkers, reflecting different redox-related pathways. 

The measure of reactive oxygen metabolites (ROM) can quantify the oxidative stress status based on the conversion of hydroperoxides to alkoxyl and peroxyl radicals under acidic conditions [6]. We previously evaluated analytical effectiveness and clinical reliability of ROM test together with the total antioxidant capacity (OXY), which can be estimated in serum samples with a colorimetric test [7,8]. 

Nitric oxide (NO) is related to endothelial dysfunction and to many CV risk factors and events (e.g., hypertension, stroke, and heart failure) [9,10,11]. NO decreased production and availability is mainly due to enhanced reactive oxygen species generation that directly inactivates NO, and induce the reduction in NO synthesis and oxidation of its receptor, soluble guanylyl cyclase [12]. As direct NO quantitative estimation is difficult, the evaluation of nitrite/nitrate (NO_x_), which are the final stable metabolites of NO, is usually done [13].

Recently, great interest was elicited by lectin-like oxidized low-density lipoprotein (OxLDL) receptor-1 (LOX-1), the major receptor for OxLDL [14]. Uptake of OxLDL through LOX-1 induces a cascade of events implicated in the pathogenesis of atherosclerosis and plaque instability and reflected by the increment of ROS, reduction of NO, monocytes recruitment, and apoptosis induction [15,16,17]. Expression of LOX-1 is induced by many inflammatory cytokines, oxidative stress, hemodynamic stimuli, and OxLDL, and it is enhanced with the presence of CV risk factors [18,19,20]. 

The aim of the study was to evaluate the levels of new recently proposed oxidative-related parameters, including ROM and OXY, in order to estimate their effectiveness as additive risk or illness indicators of stable and acute coronary events, as well as NO_x_ and LOX-1 available in a subgroup of patients. For this purpose, the correlation between oxidative stress-related biomarkers with traditional CV risk factors (aging, gender, hypertension, diabetes mellitus, dyslipidemia, smoking habits), were also assessed. Moreover, as no study has reported the response over time of oxidative stress parameters during acute myocardial infarction (AMI), we also aimed to evaluate the response of these oxidative-related parameters during the course of AMI. 

## 2. Materials and Methods

### 2.1. Patient Population

A total of 76 patients, hospitalized with a diagnosis of ACS (ST-segment and non-ST-segment elevation myocardial infarction: STEMI and NSTEMI, or unstable angina) were retrospectively enrolled in the study (ACS group). The other 77 patients, admitted to our Institute due to documented or suspected ischemic heart disease, and who underwent coronary angiography, were included as stable CAD (CAD group), and 63 non-smoker subjects nor Type 2 diabetes (T2D), and no history of past or present CAD, were considered as controls (CON group). Demographic and clinical data, admission laboratory, and instrumental parameters were collected from the Institute electronic databank. Arterial hypertension was defined when systolic blood pressure >140 mmHg and/or diastolic pressure >90 mmHg or use of antihypertensive medication, T2D when fasting plasma glucose >126 mg/dL or use of antidiabetic treatment, and dyslipidemia when total cholesterol was ≥200 mg/dL, or triglyceride ≥150 mg/dL, or current use of lipid-lowering drugs. Smoking history was coded into never smokers (who had never smoked), and smoking history (including ex and current smokers). All these variables were coded in a dichotomized classification. Exclusion criteria included major diseases like renal or liver diseases, or infectious, chronic inflammatory, or immunologic diseases or malignancies. These conditions were assessed by physical examination and routine laboratory tests. 

All subjects gave written informed consent for their participation in the study which was approved by the local ethics committee in agreement with the principles outlined in the Declaration of Helsinki. This study protocol has been approved by the local Ethical Committee, and registered to European Clinical Trials Database (EudraCT: 2009-010869-23).

### 2.2. Blood Sampling and Biochemical Analysis

Blood samples were collected immediately after admittance in all AMI patients and at 6, 12, 18, 24, 36, and 48 hours after admission (T0-T6, respectively) in an AMI subgroup. Fasting samples were collected from CAD and CON subjects in the morning. Blood samples were centrifuged at 2000× *g* for 10 min (4 °C for ROM and OXY). Serum samples were immediately analyzed or stored at −80 °C before assay (within 3 months).

We previously evaluated the analytical and clinical performance of D-Rom test (Diacron, Italy) in asymptomatic subjects and coronary artery disease patient cohorts [7,8,21,22]. In brief, this assay is based on the capacity of transition metals to catalyze peroxides in the sample and form alkoxy and peroxy radicals, which then react with an amine, leading to the production of colored species that can be spectrophotometrically detected [7]. The results are expressed as arbitrary units (AU).

OXY-Adsorbent assay (Diacron, Italy) is based on the ability of endogenous antioxidant capacity to oppose the oxidant action of added hypochlorous acid [7,23]. All standards and samples should be diluted 1:100 with distilled water before the analysis. Samples to be tested undergo the oxidant action of a known-title HClO solution, in excess respect to the ability to be adsorbed from the antioxidants present in the sample. After 10-minute incubation at 37 °C, residual HClO undergoes the reaction with an alkyl-substituted aromatic amine, leading to the formation of pink-colored species that can spectrophotometrically be detected (540 nm). The concentration of the colored complex is directly proportional to the concentration of HClO and indirectly proportional to the antioxidant capacity of the sample. Specifically, the evaluation of the absorbed quantity is obtained from the difference between the absorbance of a white reagent (constituted only by HClO) and that of the sample antioxidant capacity that buffers the oxidation induced by the acid. Sample concentration may be calculated according to the following formula:

Sample concentration = ((Blank absorbance − Sample absorbance)/(Blank absorbance − Calibrator absorbance)) × Calibrator concentration

The results are expressed as μmol of HClO consumed by 1 mL of sample (μmol HClO/mL).

At the time of NO_x_ assay, samples were ultra-filtered through 30 KDa molecular weight cut-off filters (Amicon) and centrifuged at 14,000× *g* for 10 min. NO_x_ concentration in ultrafiltrates was determined by an assay kit (Cayman, Ann Arbor, USA) based on the Griess reaction, as we previously described [13,24,25]. In brief, this colorimetric assay consists of three main steps: (1) enzymatic conversion of nitrate to nitrite by means of nitrate reductase; (2) incubation with Griess reagent for 10 min at room temperature to convert nitrite into a chromophore compound; (3) quantitative estimation of nitrite concentration by spectrophotometric measurement of the absorbance at 540 nm (ETI-system, Sorin Biomedica, Vercelli, Italy). The results were expressed as μmol/L.

The Human LOX-1 solid-phase sandwich ELISA (enzyme-linked immunosorbent assay, Thermo Scientific, Waltham, MA, USA) utilized a target-specific antibody pre-coated in the wells of the microplate, where samples, standards, or controls are then added and bind to the immobilized (capture) antibody. The sandwich is formed by the addition of the second (detector) antibody, a substrate solution is added that reacts with the enzyme-antibody-target complex to produce a spectrometrically measurable signal. The intensity of this signal is directly proportional to the concentration of target present in the samples. The results were expressed as ng/L.

Plasma high sensitivity Troponin T (hs-TnT) was performed by using ECLIA on the Elecsys automated analyzer (Roche, Basel, Switzerland), and expressed as ng/L.

### 2.3. Statistical Analysis

Kolmogorov–Smirnov test was used to assess normality distribution of each variable. Continuous data were presented as mean ± SD, unless differently specified. Categorical data are summarized as numbers (percentages). Statistical analyses included Student’s *t*-test, simple regression analysis, and Spearman’s correlation used to estimate the association between continuous variables, 𝜒^2^ tests used for comparing categorical characteristics, and analysis of variance (ANOVA) and Scheffe’s test as post-hoc analysis. Owing to skewness, log transformation of hs-TnT was used for statistical analyses. Log-transformed values were then back-transformed for data presentation.

The following variables were evaluated to assess the univariate correlation with ROM, and OXY, LOX-1, and NO_x_, in AMI patients: hypertension, dyslipidemia, diabetes, smoking history, male gender, age. Univariate predictors with a *p*-value <0.05 were entered in the multivariate model, to estimate independent predictors for ROM, and OXY after adjusting for covariates. 

A *p*-value <0.05 was considered significant and the confidence interval (CI) set at 95%. 

## 3. Results

### 3.1. Study Population Characteristics

Clinical characteristics of the overall population are shown in Table 1. Relative to the other groups, stable CAD patients were older, and with a higher percentage of males, dyslipidemia and T2D. 

### 3.2. Response over AMI Time of Oxidative Stress-Related Biomarkers

**Figure 1:**
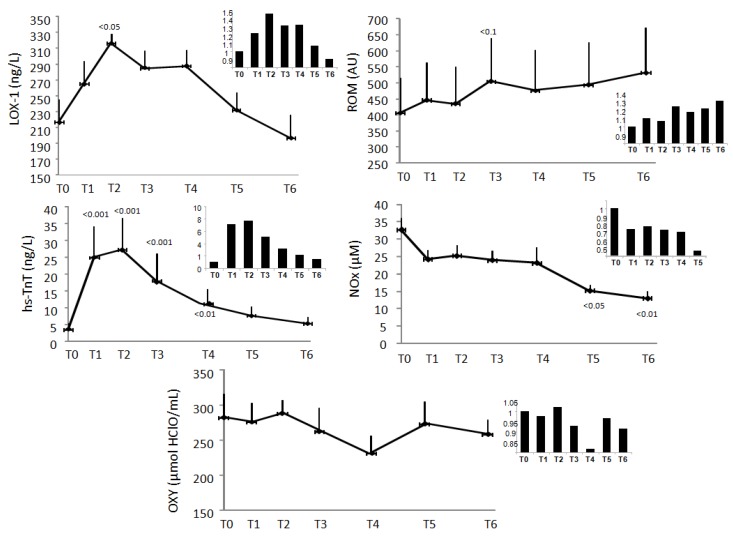
Time response during acute myocardial infarction (AMI) in 15 patients and in the small box of the figure increase ratios respect to admission values of following oxidative stress-related biomarkers; oxidized low-density lipoprotein receptor-1 (LOX-1), reactive oxygen metabolites (ROM), high-sensitivity troponin T (hs-TnT), nitric oxide (NO_x_), antioxidant capacity (OXY), respectively. Sampling points T0 were taken at admission, sampling points T1, T2, T3, T4, T5, and T6 at 6, 12, 18, 24, 36 and 48 h after admission, respectively. Results are reported as mean ± SEM. *p* < 0.1, *p* < 0.05, *p* < 0.01, *p* < 0.001 versus baseline.

In Figure 1 are reported the response over time of LOX-1, ROM, NO_x_, and OXY in 15 patients (mean and increment respect to admission, respectively). LOX-1 follows the response over time of hs-TnT, reaching maximal values at 12 h after admission (*p* < 0.05 versus T0), successively decreasing (Figure 1). Over time, ROM increased (from 406 to 531.7 AU) while NO_x_ decreased (from 32.7 to 15.2 µM) (Figure 1). OXY levels decreased, without reaching statistical significance (from 282.6 to 258.4 μmol HClO/mL) (Figure 1).

### 3.3. Reactive Oxygen Metabolites (ROM) 

**Figure 2:**
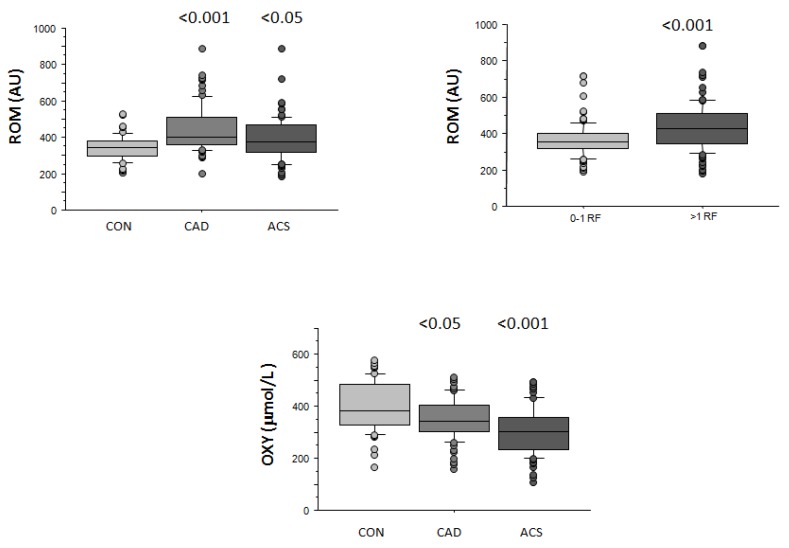
Reactive oxygen metabolites (ROM) levels in the three groups (control (CON), coronary artery disease (CAD), and acute coronary syndromes (ACS)), and according to number of risk factors (determinants included are type 2 diabetes (T2D), hypercholesterolemia, hypertension, smoking history), and antioxidant capacity (OXY) levels in CON, CAD, and ACS. Results are expressed as median and interquartile range. *p* < 0.05, *p* < 0.001 versus baseline.

CAD patients showed higher mean ROM levels with respect to ACS group and healthy controls (445 ± 126, 393 ± 116, and 342 ± 69 AU, respectively, *p* < 0.001; Figure 2). When CV risk factors shown in Table 1 were evaluated, ROM values were found higher in patients presenting dyslipidemia (423 ± 130 vs. 371 ± 95 AU, *p* < 0.001) and T2D (443 ± 127 vs. 384 ± 111 AU, *p* < 0.01) respect to those without these risk factors, and resulted higher in smokers and ex-smokers compared to non-smokers (419 ± 111 vs. 387 ± 118 AU, *p* < 0.05). 

ROM concentration also increased with the number of CV risk factors (434 ± 131 and 364 ± 90 AU, 0–1 versus ≥2 risk factors, respectively, *p* < 0.001; Figure 2).

A logistic regression analysis was applied to verify the effect of significant variables in determining increased ROM (>461 AU, 75th percentile), identifying CAD (Odds ratio, 95% CI, p; 12, 52.5–57, <0.01), ACS (6.7, 1.4–32, <0.05), and T2D (2.5, 1.2–5.3, <0.05) as factors independently associated with elevated ROM levels.

### 3.4. Antioxidante Capacity (OXY)

ACS patients showed lower OXY with respect to CAD patients and controls (304 ± 89 vs. 352 ± 77 and 394 ± 93 μmol HClO/mL, respectively, *p* < 0.001; Figure 2). OXY decreased progressively according to smoking habits (316 ± 85, and 323 ± 79 vs. 357 ± 95 μmol HClO/mL, respectively in smokers, ex-smokers, and non-smokers, *p* < 0.05), but did not correlate with levels or prevalence of the other CV risk factors, neither with the total CV risk factor number.

The multivariate analysis identifies ACS (4.6, 2.1–9.9, <0.001) as the only independent risk factor for reduced OXY (<341 µmol HClO/mL, 50th percentile).

### 3.5. Systemic Oxidative Stress Status

**Figure 3:**
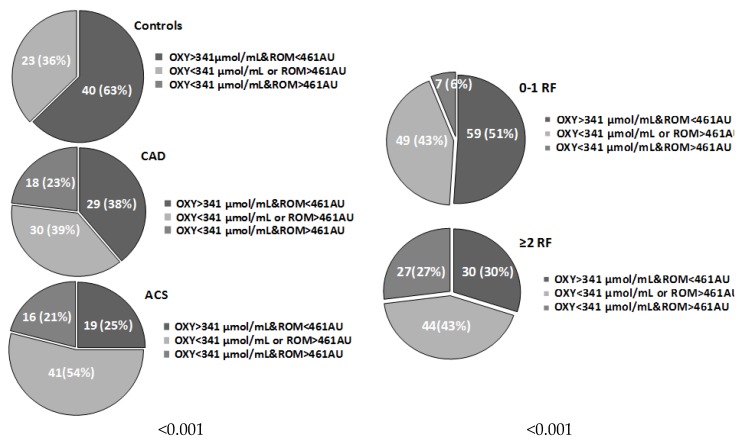
Number (percentage) of patients according to oxidative stress categories in control (CON), coronary artery disease (CAD) and acute coronary syndromes (ACS) group, and number of risk factors (Determinants included are type 2 diabetes (T2D), hypercholesterolemia, hypertension, smoking habits).

Patients in the three groups (CON, CAD, and ACS) or with different number of risk factors were stratified in line with systemic oxidative stress categories (ROM > 461 AU, 75th percentile and OXY < 341 μmol HClO/mL, 50th percentile) and reported in Figure 3 (*p* < 0.001 and *p* < 0.001, respectively). No subjects in the CON group belonged to the highest oxidative status category. The two higher oxidative stress categories were more frequent in CAD and ACS groups, and in subjects with more than one CV risk factor.

### 3.6. Nitrite/Nitrate (NO_x_)

NO_x_ was available in 50 ACS patients and 12 controls, showing a mean value of 20 ± 13 and 30 ± 11 μmol/L, respectively (*p* < 0.05). Among the CV risk factors reported in Table 1, only T2D presence resulted in associated NO_x_ values (34 ± 20 vs. 21 ± 12 µmol/L, *p* < 0.05). No association with the number of risk factors was observed.

### 3.7. Lectin-Like Oxidized Low-Density Lipoprotein Receptor-1 (LOX-1)

LOX-1 was available in 15 AMI patients, giving a mean value corresponding to 226 ± 112 ng/L. Among all variables showed in Table 1, the only parameter significantly associated with LOX-1 was aging (*r* = −0.6, *p* < 0.05).

## 4. Discussion

### 4.1. Reactive Oxygen Metabolites (ROM) and Antioxidant Capacity (OXY)

The evaluation of ROM and OXY has been largely evaluated in terms of analytical values and clinical significance (levels in general populations and cardiovascular disease patients, as well as predictors of adverse CV events in CAD) and in a different clinical setting by us and other authors [6,7,8,26,27]. For the first time, we evaluated ROM and OXY response over time during AMI, which showed a progressive increase and decrease, respectively, reflecting a progressive increment of the systemic oxidative stress status during AMI course. 

Interestingly, when both parameters were taken into account stratifying patients according to ROM and OXY categories, none of the subjects in the control group presented values in the highest category of oxidative stress, again suggesting elevated oxidative stress in ischemic disease. Nonetheless, stable CAD patients showed higher ROM values also with respect to ACS patients, implying that systemic oxidative stress status as measured by this assay likely reflects a chronic process. Instead, OXY, which shows the total antioxidant capacity, appeared progressively reduced from CON to CAD and ACS group. Different previous results suggested antioxidant consumption during AMI, especially during myocardial reperfusion injury. In particular, different vitamins (e.g., vitamins C, E, A, and β-carotene), as well as antioxidant enzymes (e.g., glutathione peroxidase), have been found markedly dropped in AMI patients [28]. Thus, findings in acute and stable CAD patients, as well as an antioxidant response over time during ACS, confirmed the chronic fall and severe acute damage to the antioxidant system in ischemic disease.

### 4.2. Nitrite/Nitrate (NO_x_)

NO_x_ are end-product of NO metabolism, and as the majority of circulating nitrite/nitrate derived from the l-arginine-nitric oxide pathway, their evaluation is a reliable indicator of NO production [29]. In our results, the reduction of NO_x_ during AMI course, suggest NO impairment during acute ischemic event. Moreover, blood NO_x_ levels, reduced in AMI patients with respect to healthy subjects, likely reflect endothelial dysfunction in this condition.

There are significant differences regarding NO_x_ levels in available studies concerning CV disease and risk. In two studies, NO_x_ levels were found to be higher in AMI and CAD patients with respect to controls [30,31]. Moreover, levels of NO_x_ appeared significantly higher in relation to our results [30,31]. In this context, it must be taken into account that the activation of inducible NO synthase (iNOS, one of the key enzymes generating NO), as a result of vascular inflammation and injury, may increase systemic NO_x_ levels, in absence of a recovery of the release of endothelial NO [32]. Moreover, variations related to methodology and difference in clinical population or study characteristics may additional contribute to these different results in terms of means. Instead, more recent data reported NO_x_ values comparable to our data in AMI patients, as well as in general populations [33,34,35]. 

### 4.3. Lectin-Like Oxidized Low-Density Lipoprotein Receptor-1 (LOX-1)

LOX-1 is highly expressed by intimal smooth muscle cells and in lipid-laden macrophages in the advanced plaques, is released in the bloodstream at the time of plaque rupture, and as such has been proposed as a marker of plaque instability [19]. Accordingly, in-house immunoassays, soluble LOX-1 (sLOX-1) emerged as a potential diagnostic marker to identify ACS at the earliest stage, and discriminate ACS without ST elevation or abnormal Q waves and ACS without TnT elevation from non-ACS [20,36,37]. Interestingly, the peak time of sLOX-1 resulted even earlier than that of TnT (around 24 h) [20]. However, in this previous study, samples were only taken at admission, and at 24 h, whereas we collected more points within this time interval, observing that systemic LOX-1 response over time follows hs-TnT trend during AMI, with a coincident peak at 12 h after admission for both sLOX-1 and hs-TnT. Nonetheless, LOX-1 increment resulted lower than the one given by troponin (about one and a half with respect to six times, respectively). Accordingly to previous data [20], we did not observe any significant correlation between sLOX-1 with TnT or C reactive protein (inflammatory biomarker) (unshown data), suggesting that sLOX-1 probably did not represent a marker for cardiac necrosis or injury or inflammation, and that all together these biomarkers may underscore different aspects of the multifaceted entity related to plaque development. In fact, primary events that cause ACS, like the rupture of plaque with the formation of a thrombus, might be better identified with the increase in sLOX-1 levels, while subsequent damage and necrosis of the heart muscle cells may be more effectively reflected by the increase in TNP concentration. We cannot say exactly when serum sLOX-1 may increase before the onset of ACS, however, levels of this biomarker were already high at patient admission respect to the last point, not excluding that sLOX-1 may begin to rise even before the onset of ACS. 

More recently, the availability of ELISA kits, like the one produced by ThermoFisher and used in the present study, may further increase reliability and reproducibility of results. Unfortunately, data of LOX-1 were available only in a few AMI patients, and we cannot compare results levels in CAD patients and controls. As a fact, we previously evaluated LOX-1 in controls and CAD, but a comparison of present results is impossible, because, at that time, LOX-1 was measured by using an in-house double-sandwich ELISA kit [38]. In general, there is still great variability between LOX-1 results, especially because ELISA in-house methods may markedly differ according to sample types, the conjugated antibody, and recognized epitopes [39,40,41,42]. 

## 5. Conclusions

The present results are in accordance with a number of previous studies highlighting higher oxidative stress status (increased ROM and decreased OXY) in both stable and acute ischemic disease compared with healthy controls, and the relationship between oxidative stress and presence and number of CV risk factors. Moreover, ACS patients showed a more deteriorated antioxidant status compared with stable CAD patients and healthy controls, suggesting that an acute coronary condition has probably insufficient time to enhance the protective responses and adaptive mechanisms.

For NOx it is necessary to better understand when systemic NO_x_ evaluation reflects endothelial impairment and vascular damage (decreased levels) or a more generalized oxidative and inflammatory status (increased levels). 

Available data suggest that LOX-1 is not a marker for cardiac necrosis or inflammation, but it may reflect additive events related to plaque instability, as it appeared significantly increased in patients at the early phase of AMI, and as such it may have a possible additional diagnostic significance as well as prognostic stratification value in AMI setting. However, in views of actual high inter-assay variability, LOX-1 measurements should be interpreted in the context of the test used, because differences between assays may have a large impact on the classification of healthy subjects and patients. The recent development of commercial ELISA may contribute to overcome these problems, although further efforts in terms of validation and standardization of the methods are needed in order to reach a consensus on assay performance. 

Data presented in this study are preliminary, and enrollment of other patients and controls is currently underway to complete and confirm present results. The applicability of oxidative stress-related biomarkers to study the pathogenesis and clinical outcome of ischemic disease derived from the undoubted pivotal role of oxidative stress in atherosclerosis. Currently, some oxidative-stress related biomarkers appear promising for future clinical use. It will be of critical importance to improve methodological and standardization issues, demonstrate the value of the novel markers over traditional risk factors in powered validation cohorts, and eventually develop an adequate multi-marker panel targeted to cardiovascular prevention, diagnosis, patient stratification, and treatment.

## Figures and Tables

**Table 1:** Characteristics of study population.

Number	CON (63)	CAD (77)	ACS (76)	*p* Value
*Age (Years)*	64 ± 7	69 ± 9	66 ± 11	<0.05
*Male gender*	25 (40)	53 (69)	36 (47)	<0.01
*Hypertension*	27 (42)	45 (59)	37 (49)	ns
*Dyslipidemia*	16 (25)	51 (67)	39 (51)	<0.001
*Type 2 Diabetes*	0 (0)	27 (35)	18 (24)	<0.001
*Smoking history*	0 (0)	31 (41)	32 (42)	<0.001

CON = controls; CAD = coronary artery disease patients; ACS = acute coronary syndrome patients. For definition of hypertension, dyslipidemia, Type 2 Diabetes and smoking history, see Material and Method section.
